# Application of the Principles of Systems Biology and Wiener’s Cybernetics for Analysis of Regulation of Energy Fluxes in Muscle Cells *in Vivo*

**DOI:** 10.3390/ijms11030982

**Published:** 2010-03-08

**Authors:** Rita Guzun, Valdur Saks

**Affiliations:** 1 Laboratory of Fundamental and Applied Bioenergetics, INSERM E221, Joseph Fourier University, 2280 Rue de la Piscine BP53X 38041, Grenoble Cedex 9, France; E-Mail: rita.guzun@gmail.com; 2 Laboratory of Bioenergetics, National Institute of Chemical Physics and Biophysics, Tallinn, Estonia

**Keywords:** muscle cells, respiration, regulation, metabolic homeostasis, mitochondria, cytoskeleton, systems biology, phosphotransfer networks

## Abstract

The mechanisms of regulation of respiration and energy fluxes in the cells are analyzed based on the concepts of systems biology, non-equilibrium steady state kinetics and applications of Wiener’s cybernetic principles of feedback regulation. Under physiological conditions cardiac function is governed by the Frank-Starling law and the main metabolic characteristic of cardiac muscle cells is metabolic homeostasis, when both workload and respiration rate can be changed manifold at constant intracellular level of phosphocreatine and ATP in the cells. This is not observed in skeletal muscles. Controversies in theoretical explanations of these observations are analyzed. Experimental studies of permeabilized fibers from human skeletal muscle *vastus lateralis* and adult rat cardiomyocytes showed that the respiration rate is always an apparent hyperbolic but not a sigmoid function of ADP concentration. It is our conclusion that realistic explanations of regulation of energy fluxes in muscle cells require systemic approaches including application of the feedback theory of Wiener’s cybernetics in combination with detailed experimental research. Such an analysis reveals the importance of limited permeability of mitochondrial outer membrane for ADP due to interactions of mitochondria with cytoskeleton resulting in quasi-linear dependence of respiration rate on amplitude of cyclic changes in cytoplasmic ADP concentrations. The system of compartmentalized creatine kinase (CK) isoenzymes functionally coupled to ANT and ATPases, and mitochondrial-cytoskeletal interactions separate energy fluxes (mass and energy transfer) from signalling (information transfer) within dissipative metabolic structures – intracellular energetic units (ICEU). Due to the non-equilibrium state of CK reactions, intracellular ATP utilization and mitochondrial ATP regeneration are interconnected by the PCr flux from mitochondria. The feedback regulation of respiration occurring *via* cyclic fluctuations of cytosolic ADP, Pi and Cr/PCr ensures metabolic stability necessary for normal function of cardiac cells.

## 1. Introduction

Cellular life depends on the availability of energy to maintain intracellular ionic homeostasis, biosynthesis and to perform the biological work, such as muscular contraction. Rapid progress in studies of complex biological systems has led to understanding of the importance of specific interactions between their components for explaining biological functions [1–5]. One of the aims of these studies – now called Systems Biology – is the quantitative description of mechanisms of interactions within systems and resulting new, system level properties, which do not exist when the components are isolated [4–6]. This approach has been successfully used in cardiac physiology [1] and is at the center of international research programs [7]. It is also a useful tool for studies of cellular mechanisms of metabolic regulation [4,6,8–11]. In our two previous reviews in the *International Journal of Molecular Sciences* [4,6] we have shown that one of these important system level properties is metabolic compartmentation, resulting from structural interactions between cellular structures and local restrictions of diffusion within organized intracellular structures. In adult muscle and brain cells metabolic compartmentation of adenine nucleotides is related to the functioning of phosphotransfer networks connecting the processes of ATP synthesis and its utilization [10–18]. Recent experimental data strongly support this conclusion, showing that the mechanism of regulation of mitochondrial respiration by mitochondrtial creatine kinase reaction *in vivo* is related to system level properties and is very different from that *in vitro*. The aim of this review article is to analyze this new data and the theoretical approaches in quantitative modeling of energy fluxes *in vivo*, with a main focus on the regulation of mitochondrial respiration in cardiac and skeletal muscle cells under normal physiological conditions when heart function is governed by the Frank-Starling law [8]. Under these conditions heart functioning is characterized by metabolic stability or homeostasis, when large-scale changes in the workload and respiration rates are observed at practically unchanged intracellular levels of ATP and phosphocreatine [19,20]. About 60 years ago Norbert Wiener developed a general cybernetic theory of feedback regulation for analysis of control and communication and applied it later to explain general mechanisms of homeostasis [21]. We show for the first time that this theory is a very useful basis for a quantitative description and analysis of complex intracellular mechanisms of regulation of metabolism and energy fluxes in the framework of Molecular System Bioenergetics, a part of Systems Biology [22].

## 2. Controversial State of the Art of Muscle Bioenergetics

In spite of intensive experimental and theoretical research, the mechanisms of intracellular regulation of energy fluxes and mitochondrial respiration in muscles *in vivo*, including heart and skeletal muscle, are still highly debated and not clear [8,11,23–32]. Controversies in this important area of physiological research is even increasing because of principal differences in points of views of authors to the nature of intracellular medium and to the state of the phosphotransfer reactions. Some authors still analyze the energy metabolism by using the quantitative theories of homogenous reaction medium without any diffusion restrictions and equilibrium state of the creatine kinase reaction [33–35]. Others, including the authors of this review, analyze the cellular energetics by taking into account the complexity of the intracellular medium, non – equilibrium state of the compartmentalized and functionally coupled creatine kinase reactions, intracellular interactions of mitochondria with cytoskeleton and other cellular structures, and local restriction of diffusion of adenine nucleotides [13,18,36,37]. The latter approach is consistent with general theories of non-equilibrium steady state kinetics of metabolic reactions described by Vellela and Qian, and dissipative metabolic structures described by De La Fuente in this Special Issue.

### Tissue Specificity of Energy Metabolism

One of the important aspects of regulation of respiration and energy metabolism *in vivo* is the tissue specificity of these processes [38]. Cardiac muscle performs permanent work requiring high energy fluxes. This is possible due to the high mitochondrial content (20–40% of cell volume) and high efficiency of extraction of Gibbs free energy for ATP synthesis (ΔG_ATP_) from the total chemical energy of free-fatty acids (FFA) or glucose. The ability of slow oxidative skeletal muscles to perform endurance work is dependent on mitochondrial content and training. In extreme cases like in the pectoral muscle of migrant birds [39] or skeletal muscles of marathon runners [40], the mitochondrial content of slow oxidative skeletal muscle is comparable to that of cardiac muscle. Endurance training, according to classical observations [41,42], induces mitochondrial biogenesis and a metabolic shift towards a more significant lipid oxidation. Only glycolytic fast skeletal muscles are characterized by low mitochondrial density, high activity of glycolytic enzymes but low energetic efficiency of anaerobic glycolysis and can only support a brief intensive exercise using the cellular stores of phosphocreatine, PCr.

Another tissue specific property of muscles is the ability of cells to preserve homeostasis of their energy metabolism *in vivo*. Measurement of phosphorus metabolites’ contents *in vivo* by biochemical analysis or by magnetic resonance spectroscopy by ^31^P (^31^P-MRS) showed that the cardiac muscle is characterized by remarkable stability of intracellular PCr and ATP contents during workload and respiration rate changes [19,20,43,44]. Metabolic homeostasis was not observed in skeletal muscles. The decrease of PCr content during muscle work and the kinetics of PCr recovery after work are dependent on the muscle type, in particular on the mitochondrial content [45]. Glycolytic muscle, where mitochondrial density is about 1% of cell volume is characterized by a rapid and deep fall in PCr concentration with a long period (about 30 minutes) of recovery of the PCr pool after the end of exercise. Slow oxidative skeletal muscles take intermediary position between cardiac and glycolytic muscles [45].

While there are remarkable agreements between experimental data from many laboratories, the explanations of this data is still very contradictory. Principal reasons for these controversies are differences in the theoretical basis for quantitative analysis of this reliable experimental data. Most clearly, these differences concern the nature of intracellular medium, state of the creatine kinase reaction, and role of the ADP – classical and uncontested regulator of mitochondrial respiration *in vitro*.

The oldest theory, but still very popular and actively used, is that of the thermodynamic equilibrium of the CK reaction in the cells *in vivo*. This theory usually assumes free ADP diffusion in the homogeneous intracellular medium surrounding mitochondria which behavior *in vivo* is taken to be similar to that *in vitro*, and allows to calculate free ADP concentration and general thermodynamic parameters such as free energy of ATP hydrolysis [24,34,35,46–54]. Concentration of cytoplasmic ADP which is in equilibrium with CK reaction in heart cells under condition of metabolic stability is about 50–100 μM [55]. This, however, leads to apparent controversy: taking into account that half - maximal respiration rate (V_max_) of isolated mitochondria (or mitochondria *in situ* surrounded by the homogenous medium) is reached in the presence of ADP in concentration of only 8–10 μM (this is the Km value for mitochondrial adenine nucleotide translocase, ANT) one can see that no regulation of respiration at cytoplasmic ADP concentration of 50–100 μM is possible, since ANT is saturated by ADP and maximal respiration rates should always be observed. This is not the case, as it is known from classical studies of Starling in heart physiology [56,57]: the respiration rate can be increased by an order of magnitude with increase of workload. Moreover, under conditions of stable ATP, PCr and creatine concentrations, characteristic for homeostasis of energy metabolism of cardiac cells, the ADP concentration should also be stable and not correspond to changes of workload and respiration rate. This is true also for inorganic phosphate, which has been taken as the main regulator of respiration [34] but which concentration is also stable under conditions of metabolic homeostasis [20]. Thus, the main counterarguments to the CK-equilibrium theory are uncontested – it is the phenomenon of metabolic stability of cardiac muscle and metabolic aspect of the Frank – Starling law of the heart [19,20,43,44,58].

In order to overcome these contradictions, to explain the regulation of mitochondrial respiration under conditions of metabolic stability but also assuming the CK equilibrium, the theory of parallel activation by Ca^2+^ was proposed and continues to be supported [23,26,59–61]. In conformity with this theory the increase of cytoplasmic Ca^2+^ during excitation-contraction coupling cycle activates ATP hydrolysis in myofibrils, and simultaneously three dehydrogenases of Krebs cycle in mitochondrial matrix increasing production of NADH and FADH_2_ by push mechanism. The oxidation of the latter increases electron flow through the respiratory chain, generating the protonmotive force and driving ATP synthesis [23,62–66]. Ca^2+^ is thought also to activate directly F_1_F_O_-ATPase and complex I [67]. However, this theory still does not fit with the requirement for the main signal of coordination of energy metabolism in cardiomyocytes recently formulated by O’Rourke [31]. According to this author the variations of cytoplasmic [Ca^2+^] have to correspond to workload and ATP consumption. However the length-dependent activation of sarcomere (mechanism on which the Frank-Starling’s law is based) is characterized by an increase in calcium sensitivity and force of contraction without any change in intracellular Ca^2+^ transients [68–70]. The Frank-Starling mechanism puts into question the viability of the theory of parallel activation of contraction and respiration by Ca^2+^. Regulation of respiration by Ca^2+^ seems to adequately explain the adrenergetic activation of oxidative phosphorylation [23,28,60,64,71–78] but not the feedback regulation of respiration by workload changes during cardiac contraction under physiological conditions of action of Frank – Starling’s law [8,79].

**Scheme 1.**
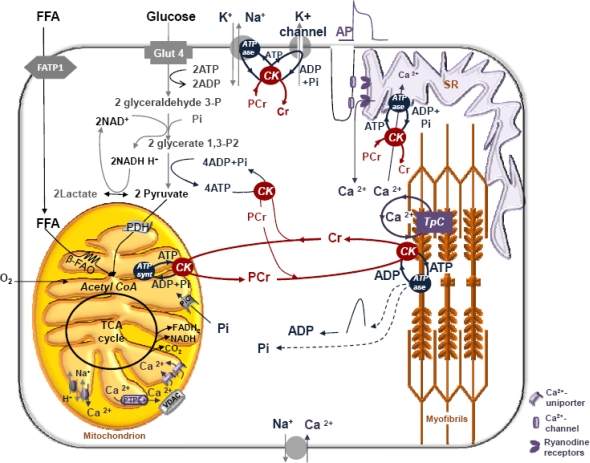
General scheme of intracellular reactions of energy metabolism in heart and skeletal muscle cells.

Both these theories assume that energy metabolism in intracellular milieu may be described as reactions in a classical homogeneous diluted solution [26,34,35,54]. This was clearly stated by Barros and Martinez [33]; this theoretical approach was criticized in one of our recent reviews [6]. Contradictions in theoretical description of cellular energetics based on CK equilibrium in an homogenous medium can be further revealed and criticized when one analyzes the works by Jeneson *et al*. [80]. In 2009, the authors published a study on the kinetics of regulation of respiration by cytoplasmic ADP evaluated *in vivo* in human vastus lateralis muscle by using ^31^P magnetic resonance spectroscopy (^31^PMRS) and mathematical modeling. The rate of oxidative ATP synthesis (Jp) was calculated from the monoexponential fit to PCr recovery (V_PCr_) after exercise and cytoplasmic ADP concentration was found from the CK reaction taken as usually to be at equilibrium. Authors found the apparent second order kinetics of the dependence of V_PCr_ on ADP concentration with Hill’s coefficient 1.9. While no direct measurements of the rates of respiration were made, the authors concluded that mitochondria *in vivo* are ultrasensitive for cytoplasmic ADP. The sigmoid kinetics of ADP/ATP exchange by mitochondrial ANT *in vivo* was proposed to explain this phenomenon [80]. This conclusion is in contradiction with all information known about the kinetics of ADT/ATP exchange by ANT [81–83], for which the mathematical model was developed by Metelkin and Demin on the basis of structural and functional data [84] that quantitatively describes the gated pore mechanism of ANT functioning. In no cases sigmoid kinetics was seen, and it is not seen when respiration is studied as the function of ADP concentration both in isolated mitochondria and in permeabilized fibers, where mitochondria are in the intracellular milieu and their behavior *in situ* can be studied experimentally (see below). Human vastus lateralis muscle contains both slow oxidative and fast glycolytic muscle fibers in almost equal amounts [85]. The latter have a very high activity of glycolytic enzymes and cytoplasmic MM creatine kinase but very low mitochondrial content [38], and inevitably glycolytic PCr production should contribute to the recovery of PCr after exercise (see Scheme 1). This was directly shown recently by Meyer’s group in studies of the kinetics of PCr recovery by ^31^P-MRS after exercise in human and rat muscles. By the end of their paper the authors correctly concluded that “using the change in [PCr] concentration during the initial period of recovery as a marker of oxidative metabolism could result in significant error if the glycolytic ATP production contribution to PCr resynthesis is not accounted for” [86]. This is in concord with the results of the classical experimental study of Kushmerick group referred to above when both ^31^P-MRS and the direct measurement of oxygen consumption was performed on isolated skeletal muscle strips [45]. Figure 5 in their paper shows that the experimentally measured respiration rates are perfectly hyperbolic and not sigmoid functions of ADP concentrations calculated from CK equilibrium. It follows from this comparison that if there is some sigmoid second order kinetics of PCr recovery with respect to apparent equilibrium ADP then it must be related to glycolytic but not mitochondrial pathway in muscle cells (Scheme 1). Slow twitch oxidative skeletal muscles mitochondria contain specific isoenzymes of creatine kinase, sarcomeric mitochondrial creatine kinase, sMtCK, a key enzyme in regulation of respiration but which was not considered at all by Jeneson *et al*. in their study [80]. These authors succeeded in reducing the behavior of the whole heterogenous complex system to that of one protein, mitochondrial ANT, a real reductionist action leading to conclusions which contradict many independent experimental data. Analysis of this situation shows that to describe the behavior of a complex system, the maximal number of interactions should be accounted for and their available quantitative characteristics should be included into models and analyzed with subsequent verification of theoretical predictions with experimental results – a Systems Biology approach is needed. In the case of muscle cell energy metabolism, modeling of both mitochondrial and glycolytic reactions and their intracellular interactions with phosphotransfer networks and cytoskeleton is needed for the correct explanation of experimental data obtained in ^31^P-MRS studies on whole muscles.

In the situation of the sharp controversies in theoretical analyses of the mechanisms of regulation of cellular energetics, there is only one way out – careful further experimental research. Experiments on permeabilized cells is very useful for solving these problems, as reviewed bellow.

Both mitochondrial oxidative phosphorylation and glycolysis are related to phosphocreatine (PCr) production. GLUT4 – glucose transporter; FATP1 – fatty acid transport protein; β – FAO – beta-oxidation of fatty acids; PHD – pyruvate dehydrogenase; PTP-permeability transition pore; VDAC – voltage-dependent anion channel; TpC – troponin C; AP – action potential. The isoforms of the creatine kinase (CK) present in different subcellular compartments are coupled with both ATP producing (mitochondrial and glycolytic) and ATP consuming (contraction, ions pumping) processes. In muscle cells sarcomeric mitochondrial CK (MtCK) functionally coupled to ATP synthase *via* adenine nucleotide translocase (ANT) and cytosolic isoformes of CK (MMCK and MBCK) coupled to glycolytic enzymes (phosphoglycerate kinase (PGK) and pyruvate kinase (PK) catalyse forward reaction of phosphocreatine (PCr) synthesis from mitochondrial or glycolytic ATP and creatine. The MMCK functionally coupled to myosin ATPases, sarcoplasmic reticulum ATPases or ions-pumping-ATPases catalyse reverse reaction of ATP regeneration from PCr and locally produced ADP. The prevalence of one of the ways of PCr production is tissue specific. In cardiac and oxidative muscle cells PCr used for muscle contraction is produced mainly from mitochondrial ATP, while in fast twitch glycolytic muscle it is produced from ATP supplied by glycolysis. Human vastus lateralis muscle is mixed muscle consisting from slow-oxidative and fast-glycolytic fibers. This is why the PCr recovery after exercise is dependent on two sources: OxPhos and glycolytic and the V_PCr_ ≠ mitochondrial V_PCr_.

## 3. Experimental Evidence for the Mechanism of Respiration Regulation in Muscle Cells *in Situ*: Human m. *vastus lateralis* and Adult Rat Heart Cells

The technique of permeabilized cells and fibers [87,88] allows us to study experimentally the mechanisms of regulation of mitochondrial respiration in muscle cells *in situ* due to preservation of the structural organization of the cells and mitochondrial contacts with cytoskeleton. Simply put, use of this method means that we can open the cell and look inside to see what really happens there and how correct our theoretical conclusions are. This is a necessary step on the half-way from the total reductionism (when isolated organelles are studied) to the systems analysis of whole intact living cells and organs (which is still too complicated for interpretation of mechanisms). During permeabilization the cytoplasmic soluble enzymes not bound to the structures, such as many glycolytic enzymes and MMCK, are released into the solution and thus no CK equilibrium exists, but the mitochondrial creatine kinase MtCK stays in the mitochondrial intermembrane space firmly fixed by lysine-cardiolipin interactions at the outer surface of the inner membrane in the vicinity of ANT [89,90]. Exogenous substrates such as ADP can be added directly to study the apparent kinetics of respiration regulation. ATP, creatine and pyruvate kinase – phosphoenol pyruvate can be added with the aim of experimental modeling of interactions between mitochondria and glycolytic system in competition for cytoplasmic ADP.

**Figure 1.**
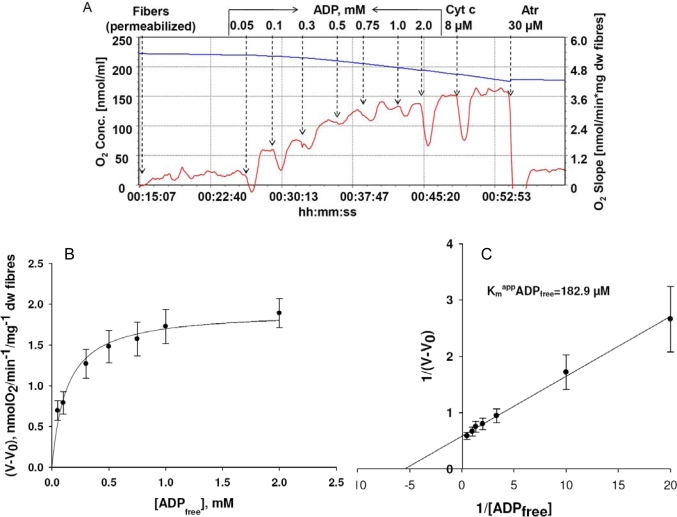
Kinetic analysis of respiration regulation by ADP in permeabilized fibres from human vastus lateralis muscle. Biopsy samples were taken from healthy volunteers and permeabilized fibers prepared as described by Kuznetsov *et al*. [88]. (A) Oxygraph recording of respiration rates during stepwise stimulation by increasing amounts of ADP in the presence of 5 mM glutamate and 2 mM malate. Cytochrome c and atractyloside are used to test the integrity of mitochondria outer and inner membranes. (B) Henri-Michelis-Menten type hyperbolic function of dependence of oxygen consumption rate on ADP concentrations. (C) Linearization of data from Figure 1B in double reciprocal plot gives the value of apparent Km for free ADP equal to 182.9 μM (n = 10). This value corresponds to mixed fibre muscle content (for comparison: in glycolytic gastrocnemius rats’ muscle the Kmapp for free ADP is about 20 μM and for highly oxidative cardiac muscle is about 300–400 μM). Reproduced from [92] with permission.

**Figure 2.**
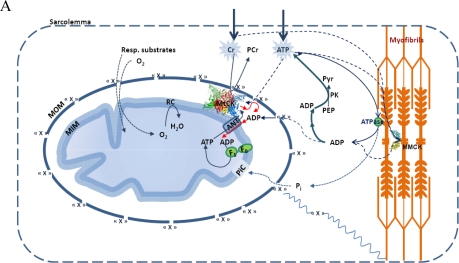

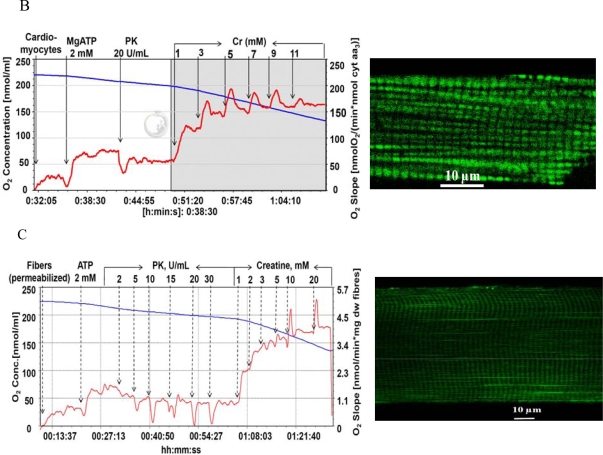
Demonstration of the central role of mitochondrial creatine kinase, MtCK, in regulation of respiration of permeabilized muscle cells. **(A)** Scheme shows the principles of the method used to study interaction between mitochondrial and glycolytic systems in competition for endogenous ADP. This Scheme shows mitochondrion *in situ* within intracellular energetic units, ICEUs [93] surrounded by cytoskeleton proteins (depicted as “x” factor) and myofibrils. Exogenous ATP is hydrolyzed by cellular ATPases into endogenous extramitochondrial ADP and inorganic phosphate (Pi). Mitochondrial (MtCK) and non-mitochondrial creatine kinases in myofibrils and at membrane of sarcoplasmic reticulum in the presence of creatine and ATP produce endogenous intra-and extramitochondrial ADP. Phosphoenolpyruvate (PEP) and pyruvate kinase (PK) system removes extramitochondrial ADP produced by intracellular ATP consuming reactions and continuously regenerate extramitochondrial ATP. Endogenous intramitochondrial ADP produced by MtCK forms microcompartments within the mitochondrial intermembrane space (IMS) and is re-imported into the matrix *via* adenine nucleotide translocase (ANT) due to its functional coupling with MtCK. Reproduced from [29] with permission. **(B,C)** Oxygraph recording of respiration of permeabilized cardiomyocytes **(B)** and fibers from human skeletal m. vastus lateralis **(C)** prepared from biopsy samples of healthy volunteers as described in Figure 1 in the presence of 2 mM malate and 5 mM glutamate as substrates. Addition of 2 mM MgATP activates respiration due to production of endogenous MgADP in ATPase reaction. Pyruvate kinase (PK) in the presence of 5 mM phosphoenolpyruvate (PEP) decreases respiration rate due to removal of extramitochondrial MgADP. Creatine in the presence of MgATP activates mitochondrial creatine kinase (MtCK) reaction of production of endogenous intramitochondrial MgADP which rapidly activates respiration up to the maximal rate (see Figure 1A), that showing that mitochondrial ADP is not accessible for PK-PEP system due to the limited permeability of mitochondrial outer membrane in the cells *in situ*. Right panels in B and C show confocal images of isolated cardiomyocytes and fibers from m. vastus lateralis, correspondingly. Reproduced from [92] with permission.

Figures 1 and 2 show the results of experiments in which the permeabilized fiber technique was used for studies of biopsy samples of human m. *vastus lateralis* taken from healthy volunteers. These experiments help us to evaluate the correctness of theoretical conclusions described above for the same human *vastus lateralis* skeletal muscle. Figure 1A shows the oxygraph recordings when ADP was added stepwise to activate the respiration to its maximal value. This data was expressed graphically in Figure 1B and linearized in double reciprocal plots (Figure 1C). The dependence of the respiration rate on ADP concentration is hyperbolic of the Henri-Michaelis-Menten type and gives in double reciprocal plots one straight line and apparent Km value for exogenous ADP equal to 183 μM, a medium value between the heart (300–400 μM) and fast glycolytic muscle fibers (about 7 μM). Similar data was earlier reported by Walsh and Sahlin [91]. The apparent Km value for ADP is much higher than found in the Kushmerick *et al*. study for cat soleus *in vivo* [45]. The reasons for this discrepancy will be analyzed below.

However, the most important data is shown in Figure 2. MgATP was first added to activate the intracellular MgATPases producing endogenous ADP that activates respiration (Figure 2B).

The PK-PEP system was then added to trap this endogenous ADP with an expected decrease in respiration rate. Finally creatine was added stepwise to study the role and kinetic of the mitochondrial creatine kinase MtCK reaction in regulation of respiration *in situ* in the situation, modeling some characteristics of intracellular milieu. Most remarkably, creatine addition rapidly activated respiration up to its maximal value (Figure 2B and 2C). In the presence of the PK-PEP system, ADP produced in MgATPase and myofibrillar MMCK reactions is trapped and rephosphorylated into ATP, and respiration is activated only due to ADP produced in the MtCK reaction. This result directly shows the central role of MtCK in the regulation of respiration in cardiomyocytes and human skelatal muscle, m. vastus lateralis, ignored by Jeneson *et al*. in their theoretical study (see above). All these experimental results described in this section and above clearly invalidate the theoretical conclusions made by Jeneson *et al*. [80].

These results, however, validate the third theory of regulation of energy metabolism *in vivo* which meets the requirements listed above and takes into account the cellular organization and multiple intracellular interactions. This is the theory of phosphotransfer networks [11,12,17,18,22]. We have shown in our previous reviews that this theory, based on experimental results from many laboratories, adequately explains the metabolic aspects of Frank-Starling’s law of the heart [8]. According to this theory, the transfer of phosphoryl groups between intracellular compartments of ATP is mainly realized *via* the PCr/CK shuttle. A small part of phosphotransfer (10–15%) can be realized by the adenylate kinase (AK) system or *via* glycolytic enzymes such as 3-phosphoglycerate kinase (PGK) or hexokinase (HK) [12,13,18,94–101]. The theory of phosphotransfer networks supposes the existence of three phenomena: the local restriction of diffusion of adenine nucleotides in the cells, functional coupling between enzymes routing phosphate metabolites and most importantly, acceptance of the fact that the reactions catalysed by CK isoenzymes are functioning in a non-equilibrium state. The phosphotransfer networks help to overcome intracellular diffusion barriers [18,97–100,102,103] and to avoid the dissipation of energy due to its transfer by ATP (ATP is used by many other intracellular reactions), and, in addition, to avoid accumulation of ADP and thus inhibition of ATPases activity and degradation of the pool of adenine nucleotides *via* adenylate kinase reaction [13]. The functioning of phosphotransfer networks is based on the principle of vectorial ligand conduction (proposed by P.Mitchell) for metabolic and transport processes within the cell [104].

## 4. Non-equilibrium Steady State of Phosphotransfer Reactions in Muscle Cells

### Origin of Controversies

Historically, the role of phosphotransfer reactions in the regulation of respiration was discovered almost simultaneously with the discovery of oxidative phosphorylation. Engelhardt found in 1930 that oxygen uptake is coupled to ATP synthesis [105]. In 1939 Belitzer and Tsybakova found that oxygen uptake in skeletal muscle homogenate is activated by creatine without addition of exogenous ADP (trace amounts of endogenous ADP present were sufficient to activate the functional coupling mechanism), and resulted in phosphocreatine (PCr) production. The effectiveness of its production measured in the homogenate of the pectoral muscle of a pigeon in the presence of creatine (PCr/O_2_) was 5.2–7 [106]. Lundsgaard showed the parallel decrease of phosphocreatine content and contractile force during long periods of muscle stimulation [107]. After that, there was a period of uncertainty: it was not clear which compound – phosphocreatine or ATP - is responsible for muscle contraction, since in physiological experiments with rapid sampling of tissue only a decrease of PCr was seen during the contraction cycle, but experiments with the actomyosin system showed that contraction needs ATP [108]. In 1950 Hill published his famous challenge to biochemistry in which he emphasized the need to find convincing evidence whether ATP or PCr was the immediate supplier of energy for contraction [109]. In 1962 Davies *et al*., solved the problem by inhibiting CK with 2,4-dinitrofluorobenzene and showing that under these conditions ATP is used during contraction [110]. This established the central role of ATP in muscle energetics, and PCr was given the modest role of an energy store used to replenish ATP at increased workloads in the CK reaction *a priori* taken to be in rapid equilibrium [108]. This was the origin of the CK equilibrium theory [108] that is still popular among many investigators as discussed above. In 1979 Veech *et al*. performed a famous study determinating of phosphorylation potential in several tissues of anesthetized rats (brain, muscle and liver) by measuring the metabolites of glyceraldehyde-3-phosphate dehydrogenase, phosphoglycerate kinase and creatine kinase, finding that all reactions are near equilibrium [111]. This result at that time was important to show that cytosolic ADP concentration is much lower than evaluated by directly measuring total ADP in tissue extracts which include also ADP bound to actin [111]. However, this important work was then enthusiastically, but without much justification, taken to show that the creatine kinase reaction is also in thermodynamic equilibrium in working skeletal muscle and heart when energy fluxes are manifold elevated. The situation for this simple theory became more complicated when the specific mitochondrial isoenzyme was discovered in Klingenberg’s laboratory [112]. Bessman, Lehninger and Jacobus, and many others, started detailed studies of the functional role of this and other CK isoenzymes (MM in muscles and BB in brain) [113–115]. Fundamental studies carried out in Theo Walliman’s laboratory gave descriptions of the structure of all creatine kinase isoenzymes and their precise intracellular localization in mitochondria, in myofibrils and at the membrane of sarcoplasmic reticulum [17,18,89,90,99,116–118]. MM creatine kinase is attached also to cardiac cells sarcolemma [97]. Such a compartmentation of CK isoenzymes and similar compartmentation of adenylate kinase led to the establishing of the theory of intracellular energy transport by phosphotransfer networks, again putting the PCr into a central position between ATP producing and consuming processes (see Schema I). In 1984 Meyer *et al*. adapted the equilibrium creatine kinase theory to this new data, showing in theoretical calculations that, because of the favorable value of the equilibrium constant, PCr may participate in energy transfer between cellular compartments [46]. This conclusion may still be valid for the cellular compartment which contains high activity of soluble MMCK, as cytoplasm of fast twitch skeletal muscles, especially for resting state. However many studies have proved, that the high efficiency of creatine control on the rate of oxidative phosphorylation is due to the functional coupling of mitochondrial creatine kinase (MtCK) with adenine nucleotide translocase (ANT) [6,10,17,18,98,99,102,119,120]. In functionally coupled systems the CK always functions in the non-equilibrium state catalyzing only one direction depending on the partners: in mitochondria ANT drives MtCK reaction in direction of PCr synthesis; in myofibrils and at cellular membranes MgATPases drive MMCK reaction in muscle cells (BBCK reaction in brain cells) in direction of local ATP regeneration at the expense of PCr [4,10,11,121]. In all cases the functional coupling includes direct metabolic channeling of adenine nucleotides or their microcompartmentation within multienzyme complexes. Non-equilibrium steady state of the MtCK reaction coupled to ANT by this mechanism is demonstrated by results shown in Figure 2B and in Table 1. While maximal activity of MtCK in forward reaction of PCr synthesis is close to that of ATP production by ATP Synthasome, coupled MtCK ensures complete utilisation of mitochondrial ATP for PCr with maximal rate and PCr/O_2_ ratio equal to 5.7 [32]. Experimental studies of kinetic properties of MtCK in isolated cardiac mitochondria show that they are dependent on the functional coupling of MtCK with oxidative phosphorylation [15,101,114,122–124]. Some authors showed that in isolated mitochondria, activation of oxidative phosphorylation decreases apparent constant of dissociation of MgATP from the MtCK-substrates complex, suggesting the privileged uptake of mitochondrial MgATP from ANT by MtCK [125]. This conclusion was directly confirmed by the isotope tracer method [126]. This high affinity of MtCK for ATP disappears when MtCK is detached from mitochondrial membranes [127]. Because of the functional coupling with ANT, the MtCK reaction mass action ratio in direction of the PCr production significantly exceeds the equilibrium constant value [128]. It has been shown in direct measurements with the ^31^P NMR inversion transfer that in different cellular compartments in hearts, the creatine kinase isoenzymes function in a non-equilibrium steady state in the direction dependent on their location and functional coupling either with oxidative phosphorylation *via* ANT in mitochondria (as MtCK), or with MgATPases in myofibrils and cellular membranes [129]. In myofibrils, the functional coupling between MMCK and myofibrillar ATPase strongly increases the turnover of adenine nucleotides at the expense of PCr [130]. In further detail, the role of coupled creatine kinase isoenzymes in phosphotransfer networks is elsewhere [16]. Only in the cytoplasmic compartment creatine kinase may approach quasi-equilibrium, especially in resting glycolytic muscles with very high cytoplasmic CK activity.

Thus, the theory of creatine kinase reaction equilibrium when it is used in many works in canonized form as discussed above is an unnecessary limitation, contradicting many experimental data and preventing us from real understanding of regulatory mechanisms in the cells. In enzymology, rapid equilibrium is considered as only a particular case of the steady state when some rate constants significantly exceed others [131]. Fortunately, in the area of theoretical studies of cellular energetics the situation is not hopeless – there is a light in the end of tunnel. There are some new promising developments in theoretical studies of cellular mechanisms of metabolic regulation. Hong Qian is developing nonequilibrium steady state (NESS) models of biological systems [132,133]; De La Fuente *et al*. are studying global self-organization of the cellular metabolic structure and formation of dissipative metabolic networks or DMNs [134]; Aon *et al*. work on development of scale-free dynamics of eukaryotic cells [135–137], and there are furthemore interesting and perspective studies in this interesting area.

## 5. Restriction of ADP and ATP Diffusion at the Level of Mitochondrial Outer Membrane–The Mitochondrial Interactosome

The maximal activation of respiration in the presence of the ADP trapping system PK and PEP (shown in Figure 2) is possible only in the case when ADP produced by MtCK is not accessible due to decreased permeability of the mitochondrial outer membrane, and is continuously recycled in the coupled MtCK – ANT – ATP Synthasome system [16,29,32]. This is not observed for isolated heart mitochondria. Because of this, the mechanisms of regulation of mitochondrial respiration *in situ* in permeabilized cardiomyocytes are radically different from those *in vitro* even if the respiration rates normalized by cytochrome aa_3_ content are comparable between them (Table 1).

The apparent Km for free ADP of isolated mitochondria surrounded by a homogeneous medium is about 7.9 ± 1.6 μM [138]. In adult cardiomyocytes, characterized by a highly structured mitochondrial network described as a “crystal-like network” [139,140], the apparent Km for free ADP is very high (370.75 ± 30.57 μM) suggesting the increased restriction of ADP diffusion at the level of the mitochondrial outer membrane. Activation of the MtCK reaction by addition of creatine induces a decrease of apparent Km for free ADP (50.24 ± 7.98 μM) due to the local ADP recycling in the MtCK reaction and its direct transfer into the matrix, itself due to the functional coupling between MtCK and ANT [138]. In non-beating HL-1 cells, derived from tumor atrial cardiac myocytes of transgenic mice and characterized by an irregular network of mitochondria in continuous fusion and fission movements [140,141], the apparent Km for free ADP (25 ± 4 μM) is close to that of isolated mitochondria suggesting the importance of the interaction of mitochondria with the intracellular environment for regulation of respiration [142]. Moreover, lysis of cytoskeleton proteins by trypsin provokes the increases of affinity of mitochondrial respiration for free ADP [143]. The apparent Km for exogenous ADP is very low in permeabilized fibers from fast twitch skeletal muscle, where it is close to that in isolated mitochondria, but high on oxidative skeletal muscle [143], and intermediate in mixed type muscle (Figure 1). Thus, all these effects are tissue specific.

The high Km value for exogenous ADP in permeabilized cardiomyocytes still puzzled Beard and Kushmerick in their recent review [27]. To find an explanation, they tried to explain our earlier observations [138] by assuming in their review that “However, it that study cell permeabilization required 30 min incubation with saponin while the isolated mitochondrial protocol involved measuring the respiration rate immediately”, and thus explaining differences in apparent Km(ADP) by the action of saponin. However, it is clearly written in that article that incubation times in both cases were identical. Moreover, saponin in these experiments is used in low concentrations and due to high affinity to cholesterol permeabilizes only sarcolemma where cholesterol content is very high [88]. Many previous studies have shown that under these conditions saponin leaves all intracellular structures intact and its effects are not time-dependent [87].

**Figure 3.**
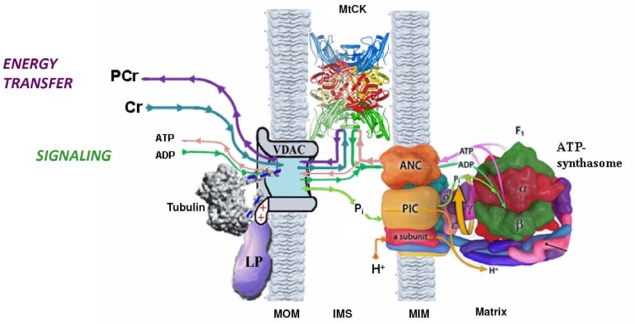
Mitochondrial Interactosome (MI) in cardiac, oxidative skeletal muscle and brain cells. Mitochondrial interactosome consists of ATP Synthasome (formed by ATP synthase, adenine nucleotide carrier (ANC) as proposed by Pedersen [152]), and inorganic phosphate carrier (PIC)), mitochondrial creatine kinase (MtCK) functionally coupled to ATP synthasome and voltage dependent anion channel (VDAC) with regulatory proteins (tubulin and linker proteins (LP)). ATP regenerated by ATP synthase is transferred to MtCK due to its functional coupling with ATP syntasome. MtCK catalyses transfer of phosphate group from ATP to creatine producing phosphocreatine (PCr) which leaves mitochondria as a main energy carrier due to highly selective permeability of VDAC. ADP is returned to and recycled in ATP Synthasome. Small signaling amounts of cytosolic ADP enter the intermembrane space (see the text) and increase the ADP recycling rate within MI maintaining increased production of the PCr. In this way coupled MtCK amplifies cytosolic ADP signal. MOM, mitochondrial outer membrane; MIM, mitochondrial inner membrane; IMS, mitochondrial intermembrane space. Reproduced from [32] with permission.

The high Km(ADP) puzzle in oxidative slow twitch muscles and cardiomyocytes was recently solved by Rostovtseva, *et al*., showing that the permeability of the mitochondrial outer membrane is dependent on interaction of its voltage-dependent anion channel’s (VDAC) with the cytoskeleton protein - heterodimeric tubulin [144–148]. Further studies of this effect led to a conclusion of existence of Mitochondrial Interactosome, as shown in Figure 3 [29,32]. This complex structure contains ATP synthasome [149–153] formed by ATP synthase, ANT and PiC, MtCK functionally coupled to ATP synthasome [98,120,121,154] and VDAC in complex with regulatory proteins such as heterodimeric tubulin and probably other linker proteins. Interactosome can include also the respiratory supercomplex [155,156]. The role of Mitochondrial Interactosome is to ensure continuous recycling of adenine nucleotides in mitochondria, their transphosphorylation and metabolic channeling of ATP *via* ANT to MtCK and ADP back, resulting in the export of the free energy from mitochondria into cytoplasm as flux of PCr. The functioning of this complex structure is best explained by the theory of vectorial metabolism and the vectorial ligand conduction, proposed by P.Mitchell, 1979 [104]. Initially, this theory was proposed to explain the organization of enzymes in super complexes allowing the scalar transport of electrons and the vectorial conduction of protons through the mitochondrial inner membrane to create the electrochemical potential [104]. Later, this concept was applied to the functioning of the phosphotransfer shuttle CK/PCr, AK [12,36,157] and to the transmission of [ADP] feedback signal from myofibril towards mitochondria [158].

A recent study of kinetics properties of MtCK *in situ* in permeabilized cardiac cells confirmed the role of mitochondrial outer membrane in the micro-compartmentation of adenine nucleotides and in the functional coupling between MtCK and oxidative phosphorylation *via* ANT in mitochondria *in vivo*, additionally showing high selectivity of this kind of control [29]. We observed the remarkable increase of the apparent constant of dissociation of exogenous ATP from binary and especially ternary complexes with MtCK *in situ* compared with isolated mitochondria (Table 2) [15,29,125]. This change of kinetics parameters suggests the decrease of apparent affinity of MtCK *in situ* for extramitochondrial ATP due to the increased restriction of MgATP diffusion through the mitochondrial outer membrane, most probably resulting from dimeric tubulin binding to VDAC. Moreover, the decrease of the apparent constant of dissociation of creatine from MtCK-substrate complexes *in situ*, in comparison with *in vitro*, suggests the increase of the apparent affinity of MtCK for creatine *in situ* (Table 2). The apparent constant of dissociation of PCr is similar for both isolated heart mitochondria and mitochondria *in situ* in permeabilized cardiomyocytes (K_ip_ de 0.89 ± 0.17 mM, Table 2). The increase of apparent affinity of MtCK *in situ* for creatine and the absence of change of apparent affinity for PCr points to the selective permeability of the mitochondrial outer membrane for these metabolites [15,29]. Parallel measurements of PCr production and O_2_ consumption in experiments with permeabilized cardiomyocytes in the presence of ADP-trapping system revealed that the PCr/O_2_ ratio (5.7 ± 0.14) is close to the theoretical efficiency of OxPhosph [29,32]. This means that PCr produced in the MtCK reaction from mitochondrial ATP is the main energy carrier out from mitochondria. Such selectivity does not exist in isolated mitochondria, in cells treated with trypsin and in non-beating HL-1 tumor cells [142,143]. The conclusion from all these studies is that the kinetic parameters of respiration regulation are system level properties dependent on complex intracellular interactions.

## 6. Role of Cytoplasmic Endogenous ADP in Regulation of Respiration

**Figure 4.**
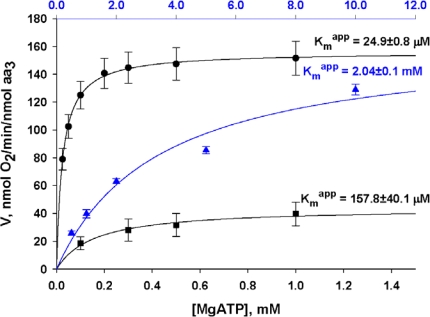
Role of endogenous ADP produced in MgATPase reactions at different added MgATP concentration in regulation of respiration of permeabilized cardiomyocytes under different conditions: (▪) – without ADP trapping system (PEP-PK) and in the absence of creatine; (•) – without PEP-PK system but in the presence of 20 mM creatine (*i.e.*, activated MtCK); (
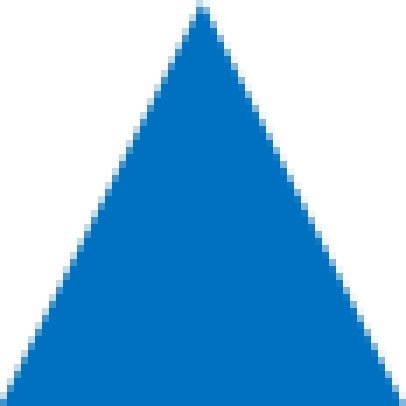
) – in the presence of both trapping system for free ADP and 20 mM creatine. Reproduced from [32] with permission.

For regulation of respiration in cardiac cells *in vivo*, the important question is that of the role of endogenous ADP produced by MgATPases [29,32]. The possible importance of this factor in the complex mechanism of respiration regulation was revealed in recent studies of kinetics of regulation of respiration of permeabilized cardiomyocytes by exogenous MgATP (Figure 4). Firstly, kinetics of regulation of respiration stimulated by endogenous ADP was studied by progressive additions of increasing amounts of MgATP; secondly, kinetics of ATP-stimulated respiration was studied in the presence of creatine, *i.e.*, activated MtCK; finally, kinetics of ATP-stimulated respiration in the presence of creatine was studied in the presence of PEP-PK system modeling glycolytic ADP consumption. In the first case respiration is stimulated by endogenous extramitochondrial ADP produced in ATPase reactions from exogenous MgATP. In the second experiment the stimulatory effect of extramitochondrial ADP is supplemented with that of intramitochondrial endogenous ADP produced in MtCK reaction. We can see that extramitochondrial ADP alone cannot effectively activate respiration. The high apparent K_m_ for exogenous ATP (157.8 ± 40.1 μM) corresponds to apparent Km of myofibrillar ATPase reaction. When oxidative phosphorylation is stimulated by both extra- and intra-mitochondrial ADP (in the presence of creatine to activate MtCK), the respiration rate increases rapidly up to maximal value and the apparent Km for ATP decreases to 24.9 ± 0.8 μM. Removal of extra-mitochondrial ADP by phosphoenolpyruvate and pyruvate kinase (PEP-PK) completely changes the kinetics of regulation of respiration. The apparent Km for exogenous ATP increases up to 2.04 ± 0.1 mM. These results show that endogenous ADP is an important regulator of respiration but only in the presence of creatine and activated MtCK. The stimulatory effect of endogenous ADP on respiration is evidently strongly amplified by functional coupling of MtCK with ANT by increasing the recycling of adenine nucleotides within Mitochondrial Interactosome.

## 7. Cybernetic Mechanisms of Feedback Regulation

**Scheme 2.**
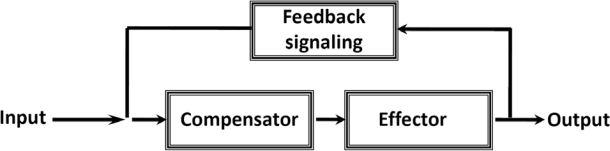
The original scheme of the general principle of feedback regulation proposed by Norbert Wiener, adapted from [21]. For explanations see the text.

Norbert Wiener was the first to introduce the principle of feedback regulation as a mechanism able to maintain homeostasis, *i.e.*, to assure stability of a system. The cybernetic theory of feedback regulation involves transmission of information which helps to make a whole of the many parts of a complex system [21]. According to Wiener two types of feedback can exist: negative and positive. “The general principle of cybernetics is negative feedback in which the required output is maintained by acting to oppose the input. Positive feedback acts to amplify the input giving a greatly increased output to any input change. This kind of regulation is generally undesirable, but being counteracted by negative feedback ensures a fast transition between an unwanted state and a desired one. The interactions of these types of feedback lead to self-limiting systems and often to cycles and oscillations in nature”. Furthermore “it may very well be that the information is carried at a very low energy level” [21]. We may adapt Wiener’s principle described in Scheme 2 for quantitative analysis of the intrinsic feedback regulation responsible for homeostasis of energy metabolism in cardiac muscle cells, if we assume the non-equilibrium state of the creatine kinase reactions in different cellular compartments and their dependence of the microenvironment – the mechanisms of functional coupling described above. For this, we need to use the mathematical model of compartmentalized energy transfer developed in several laboratories [158–160]. This model which may be called Vendelin-Aliev-Saks (or VAS model), reproducing conditions of metabolic stability and taking into account the restriction of diffusion of adenine nucleotides *in vivo*, showed the existence of oscillatory increase in ADP concentrations in the myofibril core in dependence of heart workloads. Taking into account the kinetic properties of the MMCK reaction, the model showed the formation of the cytosolic ADP signal represented by the gradient of ADP concentration between the myofibril core and mitochondrion.

### Why the Restriction of Diffusion of ADP across the Outer Mitochondrial Membrane Is Needed in Mitochondrial Interactosome?

**Figure 5.**
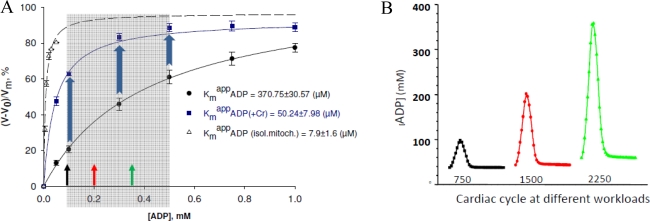
Important role of limited and selective permeability of the mitochondrial outer membrane and nonequilibrium MtCK reaction coupled to ANT in feedback metabolic signaling by cytoplasmic ADP between MgATPases and mitochondria in cardiac cells. **(A)** Dependence of respiration rate on the ADP concentration in the medium. Results of 17–29 experiments with isolated mitochondria and permeabilized cardiomyocytes are summarized. The gray area delimits physiologic range of changes in cytosolic [ADP] calculated by the model of compartmentalized energy transfer in B. In isolated mitochondria (curve (○)), no regulation of respiration is possible because of the saturating [ADP]c for the minimal workload. When the ADP diffusion is restricted as in mitochondria *in situ* in permeabilized cardiomyocytes [curve (•)], the respiration rates become linearly dependent on ADP concentrations in their physiological range. In this interval of quasi-linear dependence under physiological conditions the activating effect of ADP can be amplified by creatine [curve (▪)], due to activation of coupled MtCK. The resulting apparent Km for cytoplasmic ADP is significantly decreased and respiration rate increased. Reproduced from [92] with permission. **(B)** Calculated fluctuations of ADP concentrations in myofibrillar core corresponding to different heart workloads. Reproduced from [158–160] with permission.

By using the results of this model application for cardiac cells, we may easily understand the importance and the role of selective restriction of permeability of the mitochondrial outer membrane for regulation of respiration *in vivo* as shown by following a simple kinetic analysis. Figure 5A shows the dependence of the respiration rate of isolated mitochondria *in vitro* and in permeabilized cardiomyocytes on ADP concentration in the absence and presence of creatine, *i.e.*, activated MtCK.

When the mitochondrial outer membrane is permeable, as in isolated mitochondria, the regulation of respiration is impossible because of a saturating concentration in intracellular ADP which exceeds many times the apparent affinity of oxidative phosphorylation for free ADP (K_m_^app^ADP = 7.9 ± 1.6 μM). When the ADP diffusion is restricted at the level of MOM, as in mitochondria *in situ*, the apparent Km for free ADP becomes equal to about 370.75 ± 30.57 μM and the respiration rate becomes almost linearly dependent on cytosolic ADP concentrations (the first part of the hyberbolic curve can be approximated by linear dependence) within the interval of its values induced by workload changes, as calculated by the mathematical model of compartmentalized energy transfer, as shown in Figure 5B. Thus, cyclic changes in cytoplasmic ADP concentrations due to the non-equilibrium state of CK reactions become an effective regulatory signal, but only if creatine is present. Under physiological conditions creatine, by activating coupled MtCK within Mitochondrial Interactosome, increases respiration rate and displaces this linear dependence upward and to the left, thus amplifying the effect of cytoplasmic ADP (Figure 5A). In the presence of creatine, apparent Km for ADP becomes equal to 50.24 ± 7.98 μM. Thus, we can assume that regulation of respiration by cytosolic ADP, under condition of restriction of adenine nucleotides diffusion across mitochondrial membrane, is possible exclusively due to the specific structure of Mitochondrial Interactosome when MtCK reaction is activated and amplifies this signal due to its functional coupling with ATP Synthasome, increasing the rate of recycling of adenine nucleotides in mitochondria and the rate of respiration. This explains the metabolic aspect of Frank-Starling’s law of the heart [8,79]. This may also explain why Kushmerck *et al*. found in soleus muscle lower apparent Km for ADP than we saw in permeabilized fibers of m. vastus lateralis (Figure 1): Kushmerick *et al*. made these experiments *in vivo* where creatine was present [45], therefore they observed only the final effect of this regulatory signal and an apparent but not real Km for ADP. Kushmerick *et al*. do not need the equilibrium CK theory for explanation of their classical experimental data; this data is perfectly explained by non-equilibrium metabolic signaling.

These considerations are also important for the interpretation of the PCr recovery after exercise in oxidative and mixed type muscles, which has become an important method of *in vivo* investigation of muscle bioenergetics in health and disease [38,161–163].

## 8. Four General Characteristics of Cardiac Energy Metabolism to Be Explained by Modeling

**Figure 6.**
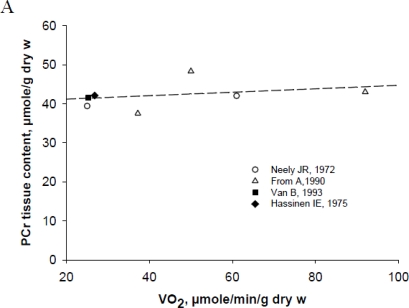

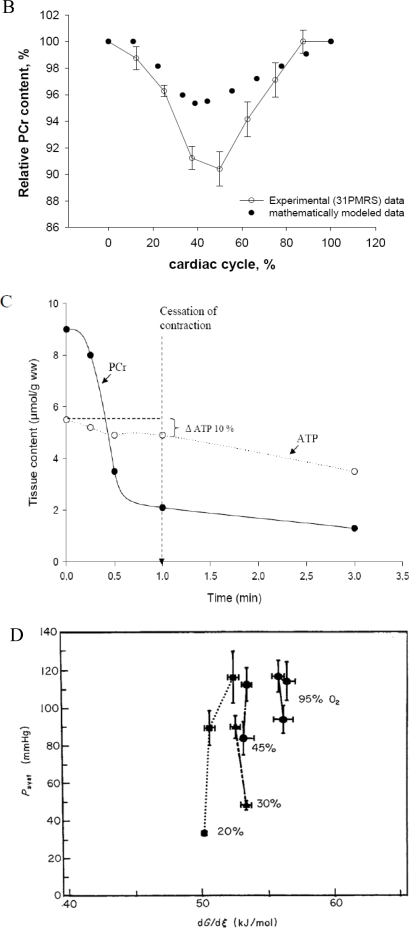
Four principal characteristics of heart energy metabolism. (A) Summary of data from different laboratories on metabolic homeostasis in heart cells. Very similar stable average intracellular PCr concentrations corresponding to different increasing respiration rates are always observed. (B) shows oscillations of relative PCr content during cardiac cycle in perfused rat’s heart under conditions of metabolic stability. The curve marked by empty circles (○) is refitted using experimental data received by Honda *et al*., 2002 in gate-pacing in [165] (with permission), only the mean values of metabolites are shown. (C) The origin of the problem of the compartmentalization of adenine nucleotides and metabolic energy sensing in cardiac cells. The data show metabolic changes in the totally ischemic dog hearts. Data are redrawn from [10] (with permission). (D) Peak systolic pressure P_syst_ of isolated isometric hypoxic rat hearts is independent from free energy of ATP hydrolysis, dG/dξ calculated from creatine kinase equilibrium and total tissue contents of creatine, phosphocreatine and ATP. Reproduced from [167] with permission.

The predictions from the mathematical modeling of feedback mechanisms of regulation of mitochondrial respiration in cardiac cells are consistent with the results of *in vivo* studies of isolated and perfused rat’s heart by using the pacing-gated ^31^PRMS [164,165]. These studies, not very often discussed, showed that under conditions of metabolic homeostasis [meaning constant average values of high energy phosphate metabolites (Figure 6A)] cardiac contraction cycles are associated with small-scale oscillations in ATP, PCr and Pi concentrations, depending on heart workload and the intrinsic kinetic properties of myosin (Figure 6B). These are two important characteristics of cardiac energy metabolism under normal conditions. Oscillations of metabolites’ concentrations were consistently showed only for *in vivo* experiments by applying sensitive techniques of synchronization with cardiac cycles but rarely accounted for in the explanation of metabolic regulatory mechanisms. The experimentally observed oscillations of phosphate metabolites are even bigger than the oscillations mathematically predicted by the VAS model (Figure 6B). For example, the amplitude of oscillations of PCr is 9.6, 1.3% for experimental and about 5.4% for mathematical models. The fitting of these oscillations by results of modelling may show the reliability of the model. However, this needs further development to achieve quantitative fitting with experimental data.

Two other important characteristics of cardiac metabolism have been revealed in the pathological conditions in classical experiments separately by Neely [19], Gudbjarnason [166], Kammermeier *et al*. [167,168] and confirmed in numerous studies [30]. Firstly, in total ischemia (no oxygen supply and perfusion to remove metabolic products), the PCr content decreases very rapidly, in parallel with contractile force when there are minor changes in the total ATP content (Figure 6C). Kammermeier group found that in a similar way, under hypoxic conditions, contractile force can be decreased manifold without any changes in the free energy of ATP hydrolysis, or phosphorylation potential (reproduced in Figure 6D). These classical data leaves no room for explanation of the regulation of ATP synthesis and hydrolysis by the mass action ratio of the ATPase reaction (free energy of ATP hydrolysis is calculated from this ratio), recently made on the basis of calculations by mathematical modeling assuming CK equilibrium and homogenous reaction medium [34,35]. This again shows that it is useless to apply the total content of ATP and derived parameters obtained on the bases of assumption of creatine kinase equilibrium as quantitative indices of cardiac function and regulation of mitochondrial respiration. All this data cannot be explained without an assumption of the compartmentalization of adenine nucleotides in the cells, according to which local concentrations of ATP regenerated at the expense of phosphocreatine in compartmentalized creatine kinase reactions are principally important for regulation of cellular function [11,17,22]. This conclusion is confirmed by results of many groups which have studied ATP fluxes in the failing human heart and diseased muscles [30,169–174].

Robert Weiss *et al*. have applied image-guided ^31^P Magnetic Resonance Spectroscopy (MRS) to study the CK fluxes in normal, stressed and failing human hearts to find that the ATP flux through CK system is reduced by 50% in absence of reduction of ATP stores [169]. Neubauer *et al*. found in systematic studies of patients with dilated cardiomyopathy by MRS that the PCr/ATP ratio at almost constant ATP stores is an index of mortality [174]. Both groups explained their data by local regeneration of ATP *via* creatine kinase reactions. James Weiss *et al*. reviewed all data showing the importance of modular spatially compartmentalized networks in heart metabolism and the need for a systemic approach for understanding of cardiac metabolism [170]. The role of changes of compartmentalized energy transfer in pathogenesis of cardiac and skeletal muscle diseases have been recently reviewed by Ingwall [30,171,172] and Ventura-Clapier *et al*. [173,175,176].

## 9. Feedback Regulation of Respiration: Application of Cybernetic Principles

**Figure 7.**
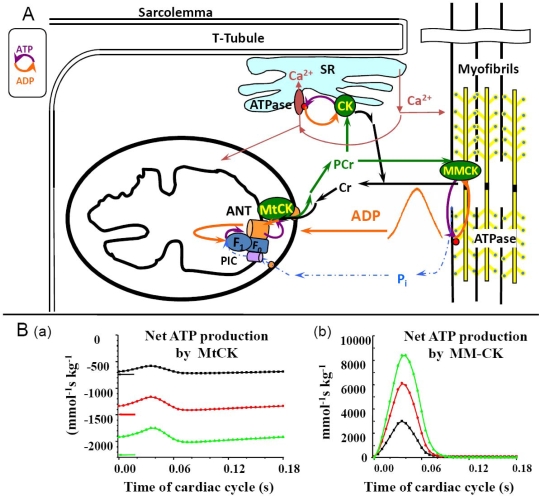
Separation of energy transfer by PCr flux and information transfer by low amplitude metabolites’ concentrations in cardiomyocytes: realization of cybernetics principle of feedback regulation. **(A)** Scheme represents intracellular energy unit (ICEU) consisting of ATP production sites (mitochondrion, glycolysis), ATP consumption sites (myosin ATPase, SERCA, ions-pumping-ATPase) communicating *via* the system of compartmentalized creatine kinase (CK) isoforms and PCr/CK phosphotransfer network. **(B)** Non-equilibrium state of MtCK in mitochondria and MM-creatine kinase reactions at sites of ATP utilization functioning in mitochondria opposite directions. **(a)** In mitochondria the constant rate of net PCr production in non- equilibrium steady state of coupled MtCK reaction is established for any workload to meet the cellular energy requirements [colored lines correspond to different workloads depicted in Figures 5B and 7B(b)]. **(b)** The cyclic changes in rates of ATP regeneration in non-equilibrium myofibrillar MMCK reaction during contraction cycles at different workloads correspond to oscillations of [ADP]c described in Figure 5B (colored lines in both figures show the amplitudes of ADP and ATP productions to different heart workloads, Figure 5B). Reproduced from [158–160] with permission.

The oscillations of ATP and PCr contents under conditions of metabolic homeostasis (as described above), give us the possibility to adapt the principle of feedback control proposed by Norbert Wiener (1947) for explanation of the mechanisms of regulation of mitochondrial respiration *in vivo* in cardiomyocytes (Figure 7). ATP hydrolysis results in the cyclic increase in local ADP concentration ([ADP] impulse) together with releases Pi in the myofibrils. The Pi is not consumed in the MMCK reaction and diffuses freely to enter the mitochondrial matrix *via* its carrier (PIC). The increase in local ADP concentration activates the reverse non-equilibrium MMCK reaction of ATP regeneration in myofibrils, and at the same time forming a gradient of ADP concentration transmitted towards the mitochondria. This cytosolic signal represented by cyclic changes in ADP concentration is amplified in mitochondria by the mechanism of functional coupling of MtCK with ATP Synthasome, as described above in detail. The intracellular concentration of creatine is about 10 mM and is greater than the K_m_ of MtCK for creatine which is *in vivo* only about 2 mM [29]. Saturating concentrations of this substrate (creatine) allows coupled MtCK to respond effectively and rapidly to the increase in supply of second substrate - mitochondrial ATP by ANT. The rephosphorylation of ADP in MMCK reaction decreases the PCr/Cr ratio. This positive feedback signal is also transferred towards MtCK *via* cytoplasm. Due to continuous recycling of ADP and ATP in coupled reactions in Mitochondrial Interactosome, mitochondria maintain constant steady state rate of production of the PCr, and thus the energy flux depending on the workload (Figure 7). The efficiency of PCr production in MtCK reaction as it was shown above is close to the maximum efficiency of oxidative phosphorylation. Thus, the coupled MMCK reaction in myofibrils and coupled MtCK reaction in mitochondria run in a non-equilibrium state in opposite directions, resulting in the separation of energy fluxes (mass and energy transfer by PCr) and signaling (information transfer by oscillations of cytosolic ADP concentrations, Pi and PCr/Cr ratio) amplified within Mitochondrial Interactosome.

**Scheme 3.**
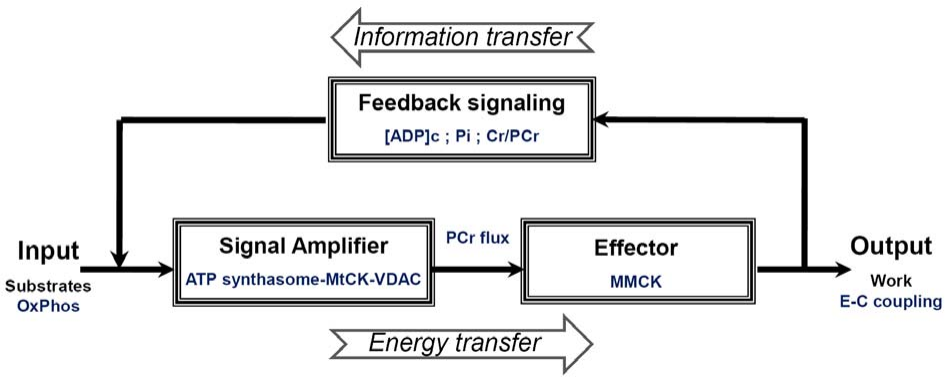
General presentation of the nature of the mechanism of feedback metabolic signaling in regulation of energy metabolism within intracellular energy units. Due to the non-equilibrium steady-state MtCK and MMCK reactions intracellular ATP utilization (marked as output) and mitochondrial ATP regeneration (marked as input) are interconnected *via* the cyclic fluctuations of cytosolic ADP, Pi and Cr/PCr.

The separation of energy and information transfer is illustrated by general Scheme 3. This Scheme shows feedback regulation of respiration *in vivo* corresponding to the cybernetic principles: the usage of ATP (or release of free energy of ATP hydrolysis (ΔG_ATP_) to perform work, marked as output) and the ATP regeneration (or extraction of ΔG_ATP_ from substrates by oxidative phosphorylation; corresponding to input). These are interconnected *via* the feedback signaling through oscillations of cytosolic concentrations in ADP, Pi and Cr/PCr. This feedback regulation of respiration ensures metabolic stability necessary for normal heart function.

Both the experimental studies of mitochondria *in vivo* and the modeling of compartmentalized energy transfer have shown that the important component of feedback metabolic signaling from ATPases to mitochondria is Pi [26,177–179]. Interestingly, this conclusion has been made even by applying the theory of CK equilibrium [34,35,54]. The flux of Pi from ATPases to mitochondria represents both mass and information transfer to satisfy the stoichiometry of ATP synthesis from ADP and Pi. While the phosphoryl group and energy are transferred from mitochondria by PCr, Pi diffuses back to mitochondria for rephosphorylation both as metabolic flux (mass transfer) and as a feedback signal (information transfer). Under conditions of metabolic homeostasis and CK equilibrium, both average values of ADP and Pi concentrations are constant and their role in respiration regulation seems to be excluded as not satisfying the O’Rourke principle (see above). Only the non-equilibrium kinetics of reactions and cybernetic principle, described above, reveals the cyclic changes in both parameters, as well as in PCr/Cr ratio, helping us to understand the feedback regulation of respiration in accordance with O’Rourke’s principle.

Thus, compartmentalized and coupled isoforms of creatine kinase functioning in a non-equilibrium state behave like Maxwell’s demons in cellular microcompartments [10] and help to maintain the intracellular homeostasis of energy metabolism in cardiac cells. In his book Norbert Wiener writes: “the amount of information in a system is a measure of its degree of organisation, entropy of a system is a measure of its degree of disorganization; and the one is simply the negative of the other” [21].

## 10. Conclusions

Quantitative modeling is a powerful method of Systems Biology and particularly very useful for research of integrated cellular metabolism in Molecular System Bioenergetics. However, for effective use of this method most important are theoretical principles upon which the models are based, and availability of reliable experimental data necessary for model construction and verification. Careful analysis shows that simple theories of reaction equilibrium in homogenous medium do not allow describing adequately the mechanisms of regulation of cellular integrated metabolism, in particular the role of creatine kinase system in muscle cell energetics and mechanisms of regulation of respiration *in vivo*. Non-equilibrium steady state kinetic analysis is a necessary as general theoretical basis of modeling. Very useful for analysis of the role of compartmentalized non-equilibrium, functionally coupled creatine kinases in muscle energetics is application of the cybernetic principle of feedback regulation developed by Wiener. We have analyzed the problems of quantitative modeling of only one of the processes of integrated energy metabolism of muscle cells shown in Scheme I – phosphotransfer reactions and feedback signaling in respiration regulation. It will be most important to integrate this pathway into more general models of energy metabolism, including all mitochondrial reactions, fatty acid oxidation, adenylate kinase shuttle, glycolysis, membrane ATPases and K_ATP_ channel with coupled creatine kinases (see Scheme I) taking into account the complex structure of cell interior and diffusion restrictions. It is still a real challenge to model mathematically the functioning of Mitochondrial Interactosome and coupled creatine kinases in other intracellular locations – to model Maxwell demons in action in the cells. This hard work in future may some days give us the “general equation of muscle cell,” using the expression of Claude Bernard [180] - a correct and complete mathematical model of its integrated metabolism.

## Figures and Tables

**Table 1.** Basic respiration parameters of isolated rat heart mitochondria and of mitochondria *in situ* in permeabilized cardiomyocytes.V^3^–respiration rate in the presence of 2 mM ADP, V^Cr,ATP^–respiration rate in the presence of activated MtCK by 2 mM ATP and 20 mM Creatine; Reproduced from [29] with permission.

**Parameter**	**Mitochondria*in vitro***	**Mitochondria*in situ*(permeabilized cardiomyocytes)**

V_0_, nmolO_2_·min^−1^·mg prot^−1^	26.37 ± 7.93	7.53 ± 1.61
V^3^_(2 mM ADP)_, nmolO_2_·min^−1^·mg prot^−1^	187.94 ± 40.68	84.45 ± 13.85
[Cyt aa_3_], nmol·mg prot^−1^	1.00 ± 0.04	0.46 ± 0.09
V^3^_(2 mM ADP)_, nmolO_2_·min^−1^·nmol cyt aa_3_^−1^	187.94 ± 40.68	178.23 ± 33.96
V^Cr,ATP^, nmolO_2_·min^−1^·nmol cyt aa_3_^−1^	197.90 ± 31.86	162.63 ± 26.87

**Table 2.** Kinetic properties of MtCK *in situ* in cardiomyocytes. Values of constants for isolated mitochondria are taken from literature [22]. In isolated mitochondria the oxidative phosphorylation decreases dissociation constants of MgATP from MtCK-substrates complexes suggesting the privileged up-take of all ATP by MtCK. In mitochondria *in situ* in permeabilized cardiomyocytes the increase of apparent constants of dissociation of MgATP compared with *in vitro* mitochondria shows the decrease of apparent affinity of MtCK *in situ* for extramitochondrial MgATP. The decrease of apparent constants of dissociation of creatine from MtCK-substrates complexes suggests the increase of the apparent affinity of MtCK for creatine *in situ*. The apparent constant of dissociation for PCr did not change *in situ* compared with isolated mitochondria. Reproduced from [29] with permission.

		**K_ia_ (MgATP)**, **mM**	**K_a_ (MgATP)**, **mM**	**K_ib_ (Cr)**, **mM**	**K_b_ (Cr)**, **mM**	**K_ip_ (PCr)**, **mM**
Isolated Mitoch.	−OxPhosph	0.92 ± 0.09	0.15 ± 0.023	30 ± 4.5	5.2 ± 0.3	
	+OxPhosph	0.44 ± 0.08	0.016 ± 0.01	28 ± 7	5 ± 1.2	0.84 ± 0.22
Mitoch*. in situ (PEP-PK)*		1.94 ± 0.86	2.04 ± 0.14	2.12 ± 0.21	2.17 ± 0.40	0.89 ± 0.17
